# Marginal Asthma Prevalence from NO_*x*_ Emissions (MANE): A Model to Predict Pediatric Asthma Burden from Emissions of Nitrogen Oxides

**DOI:** 10.1021/acs.est.4c09012

**Published:** 2025-05-19

**Authors:** Brian M. Gentry, Allen L. Robinson, Peter J. Adams

**Affiliations:** † Department of Engineering and Public Policy, 6612 Carnegie Mellon University, 5000 Forbes Ave., Pittsburgh, Pennsylvania 15213, United States; ‡ Department of Atmospheric Sciences, 3447 Colorado State University, 200 W. Lake St., Fort Collins, Colorado 80521, United States

**Keywords:** pediatric asthma, nitrogen oxides, nitrogen dioxide, environmental justice, reduced-complexity model, air quality, traffic-related air pollution

## Abstract

Pediatric asthma affects roughly 1 in 15 children in the U.S., with significantly higher rates in Black children. Exposure to NO_2_ contributes to asthma development, but quantitative analysis of pediatric asthma burden attributable to NO_*x*_ emissions is rarely performed due to a lack of suitable models. This paper describes a new model, Marginal Asthma prevalence from NO_*x*_ Emissions (MANE), which assesses the pediatric asthma burden from NO_*x*_ emissions and its distribution across race/ethnicities. We find that emissions in more densely populated areas cause a larger number of pediatric asthma cases and tend to disproportionately impact minority communities. We applied our model to assess the pediatric asthma burden from sources of NO_*x*_, finding that diesel heavy-duty vehicles are responsible for approximately 4% of all pediatric asthma cases in the U.S. Additionally, we find that all source sectors considered disproportionately impact children of color, with 65–100% higher per-capita asthma rates in Black children compared to white non-Hispanic children. Finally, we find that emissions in a limited number of urban areas are responsible for a large share of asthma cases, suggesting that local, targeted restrictions on NO_*x*_ emissions may provide great public health benefit nationally.

## Graphical abstract

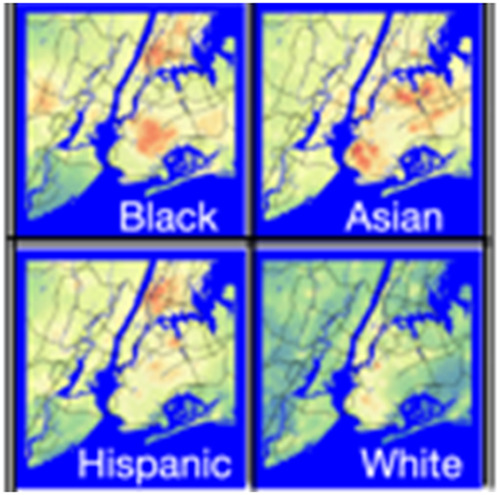

## Introduction

Pediatric asthma is a disease that affects roughly 1 in 15 children in the U.S.,^1^ causing chronic breathing problems that may persist throughout one’s life, making control of asthma rates a key focus for public health policy-making. Additionally, rates are substantially higher in children of color, particularly Black children,^2^ introducing concerns of racial equality when designing policies to reduce asthma prevalence. While some factors contributing to asthma development are uncontrollable, such as genetic predisposition,^3^ others, such as exposure to environmental hazards, are controllable. For example, exposure to nitrogen oxides (NO_*x*_), especially nitrogen dioxide (NO_2_),^4–9^ is linked with increased asthma prevalence. According to best epidemiological estimates, approximately 20% of all pediatric asthma cases in the U.S. can be attributed to NO_2_ exposure,^10–13^ and the NO_2_-attributable percentage is substantially higher, up to 30%, in major metropolitan areas such as New York or Los Angeles.^14^

Controlling NO_2_ exposure is thus a policy goal from both a public health and environmental justice perspective. However, assessing *a priori* the impact of a potential policy intervention regulating NO_*x*_ emissions on asthma prevalence is a difficult problem. Regulations limit emissions of NO_*x*_, while the health impacts are associated with concentrations of NO_2_. The standard model for estimating concentrations of air pollutants from emissions is the chemical transport model (CTM), a resource- and experience-intensive model that is ill-suited for policy screening purposes.

Reduced-complexity air quality models simplify the prediction of health impacts from perturbations in air pollutant emissions; however, most reduced-complexity models in the air quality literature focus exclusively on premature mortality from exposure to fine particulate matter,^15–18^ neglecting other pollutants. Of the reduced-complexity models in the literature, only the models Air Pollution Emission Experiments and Policy analysis (APEEP) and Co-Benefits Risk Assessment (COBRA) assess asthma-related health end points, but neither assesses changes in asthma prevalence and additionally does not do so at a high enough spatial resolution to accurately identify differences in asthma health end points by race/ethnicity.^13,19^ A standing executive order from the Clinton Administration requires federal agencies, including the Environmental Protection Agency (EPA), to consider environmental justice when developing regulations.^20^ However, there is little substantive analysis done by the EPA to quantify environmental injustice, largely due to a lack of efficient tools to analyze the impacts of perturbed emissions inventories.

There have been various efforts in the literature to attribute pediatric asthma prevalence to source sectors of NO_*x*_. Spencer-Hwang et al.^21^ investigated the correlation between asthma-related emergency room visits and proximity to rail yards, finding a statistically significant association, and Gruenwald et al.^22^ attributed nearly 13% of all pediatric asthma cases in the U.S. to NO_*x*_ emissions from gas stove cooking. While these efforts contribute to our understanding of the relationship between asthma prevalence and sources of NO_*x*_, they are not generalizable for other sources of NO_*x*_; there does not currently exist a streamlined approach to estimate the number of asthma cases associated with a particular source of NO_*x*_ nor their distribution across race/ethnicities, preventing the exploration of policy approaches to reduce asthma prevalence.

To meet this need, we developed a high spatial resolution model to predict changes in asthma prevalence from perturbations in emissions of NO_*x*_, naming our modeling approach “Marginal Asthma prevalence from NO_*x*_ Emissions,” or MANE. We combine a Gaussian plume model, AERMOD, combined with population data and epidemiological data relating changes in NO_2_ concentrations to changes in asthma rates, to predict the linear sensitivity of health end points, particularly total national asthma prevalence and prevalence by race/ethnicity, to perturbations in NO_*x*_ emissions at Census block group resolution. We then use this model to estimate the number of asthma cases and distribution across race/ethnicities attributable to major source sectors of NO_*x*_.

## Methods

### Model Development

We developed a model, MANE, that predicts the linear sensitivity of national asthma prevalence and prevalence by race/ethnicity to perturbations in NO_*x*_ emissions for a given source of NO_*x*_ emissions. MANE predicts these health end points for more than 40 million source locations, spaced on a 500 m grid covering the contiguous U.S. We first provide a high-level overview of MANE’s modeling methodology, followed by more specific modeling details.

MANE’s modeling framework involves three steps to predict changes in asthma prevalence from changes in NO_*x*_ emissions for a given source location: source-receptor air quality modeling to predict annual-average NO_2_ concentrations at evenly spaced receptors originating from a source of NO_*x*_; combination with population under age 18 and baseline disease prevalence at each receptor; and aggregation to estimate the cumulative health effect of the NO_*x*_ emissions. Each of these steps is standard in developing reduced-complexity air quality models, but as of yet such an effort has not been undertaken to predict pediatric asthma prevalence from NO_*x*_ emissions. An overview of these steps is given in Figure 1.

**Figure 1:**
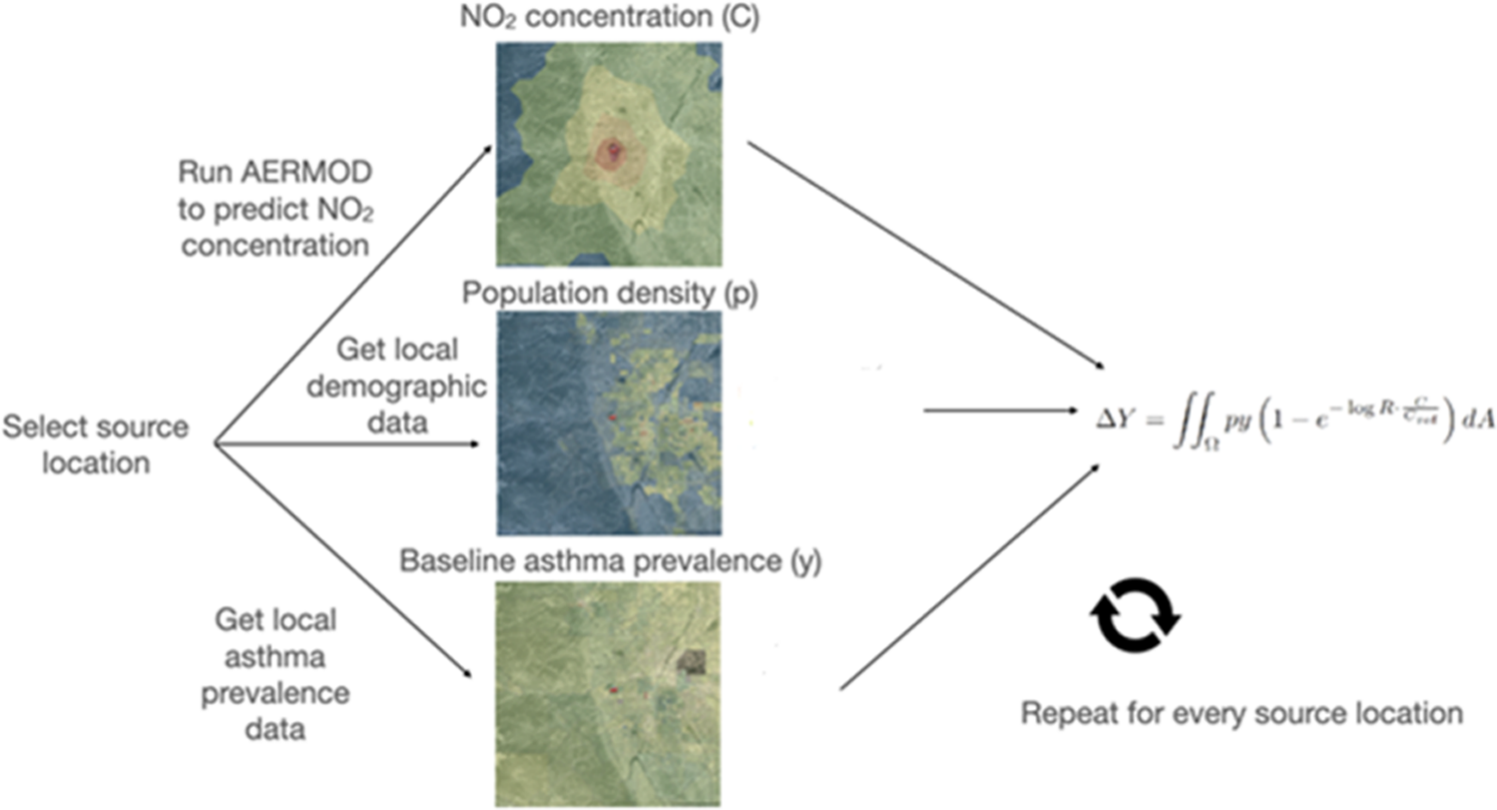
Overview of MANE modeling framework.

For the first step, we use the Gaussian plume model, AERMOD, to predict marginal changes in annual-average NO_2_ concentrations at gridded receptors from a source of NO_*x*_ with unit emissions. We selected AERMOD due to its acceptance in the regulatory community, low computational cost, and high fidelity of predicting air pollutant concentrations. We predict NO_2_ concentrations on a rectangular grid with 500 m spacing out to 72 km from the source, doing so separately for each of the 40 million source locations across the contiguous U.S. To reduce the number of AERMOD simulations required, we leverage an “average plume” approach. In each region of the U.S. in Figure S1, which we term “meteorological regions,” we use the same plume for each source located in that region, simply translated in space to be centered on each respective emissions source. These meteorological regions are, on average, of size 108 km × 108 km. This method assumes that local meteorology and terrain effects do not significantly affect predictions of sensitivities of health end points to changes in emissions. The same basic approach is used in the EPA’s National Air Toxics Assessment as well as our previously published model EASIUR-HR.^23^ This approach allows us to run AERMOD one single time for a single source in each meteorology region, after which we use that same plume for every other source in the same region.

We have previously evaluated the uncertainty introduced by this simplification for premature mortality associated with exposure to PM_2.5_, finding that this assumption introduces approximately 10% random error in predictions of cumulative health impacts and 30% random error in predictions of disparate impacts by race/ethnicity but with no bias in either. We did so by calculating premature mortality incidence from perturbations of primary PM_2.5_ emissions at more than 250,000 source locations using two AERMOD simulations, one with meteorology data from the nearest station and one with meteorology data from a different, nearby station, calculating the difference between the two. This relatively low random error is due to the fact that while plume shapes change with changing meteorology and terrain, the underlying population distribution does not. Populations near a source location are most impacted, and so while changing meteorology and terrain changes downwind concentrations, the highest concentrations near the source remain largely unaffected, producing a similar net effect with different sets of meteorology or terrain data. This is true for primary PM_2.5_, which displays very sharp concentration gradients near the source; for NO_2_, which displays shallower concentration gradients near the source (as it is largely a secondary pollutant formed via reaction of NO and O_3_), this same trend holds. While the error for a single source location can be significant, this random error diminishes when considering changes in emissions at each of the 40 million source locations (for example when one does a national analysis), as random errors from each emissions source tend to cancel as the effects of many such sources are aggregated. We do not recommend using this approach when doing analysis on a small number of source locations.

NO_*x*_ emissions are predominantly in the form of NO, which are then converted by atmospheric chemistry to be predominantly NO_2_ in the ambient. To simulate the interconversion of NO and NO_2_, we use the built-in Plume Volume Molar Ratio Method (PVMRM) in AERMOD.^24–26^ PVMRM assumes that conversion of NO to NO_2_ is ozone-limited, such that the NO_2_/NO_*x*_ ratio at any point in the plume is equal to the O_3_/NO_*x*_ ratio plus the in-stack NO_2_/NO_*x*_ ratio, which we assume to be 0.1, typical of emissions from internal combustion engines.^27^ PVMRM has been extensively validated and shows good agreement with experimental data.^24^ We input seasonal-average ambient ozone concentrations from the meteorology station used to generate the average plumes for each meteorology region. We also tested the sensitivity of our predicted metrics (described in the following paragraph) to input ozone concentrations, results that are presented in Section S3.

Using the predicted high-resolution NO_2_ concentration fields, we proceed to the second step, combining predicted concentrations with gridded population and baseline risk to predict the change in asthma prevalence and prevalence by race/ethnicity at each receptor. The relationship between changes in asthma prevalence and changes in NO_2_ concentrations is shown in eq 1, where Δ*Y* represents the change in asthma prevalence, Ω is the domain of integration (a circle with 72 km radius centered on the source), *p* is the population density under age 18, *y* is the baseline pediatric asthma rate, *C* represents the change in NO_2_ concentration, and *R* is a unitless quantity called the risk ratio associated with a reference concentration *C*_ref_, here assumed to be 1.05 and 2.1 ppb, respectively, based on a synthesis of epidemiological estimates.^14^ We obtained population data from the 2020 Decennial Census at the Census block group resolution^28^ and baseline pediatric asthma rate data from the Center for Disease Control and Prevention’s 2022 PLACES data set at the zip code resolution.^29^ Note that the modeling framework we developed can easily be updated to use more recent population or disease prevalence data. To estimate the change in asthma prevalence for a specific race/ethnicity, we only modify the population variable *p* to be the population density under age 18 of that race/ethnicity, assuming that both the baseline risk at a gridded receptor and the risk ratio remain constant across race/ethnicity; discussion of the implications of this assumption is provided in Section S1. To allow for easy comparison across race/ethnicity, we normalize the change in asthma prevalence for each race/ethnicity by the total, national population of that race/ethnicity, producing a per-capita estimate of marginal asthma prevalence by race/ethnicity.

1 $$\Delta Y=\iint_{\Omega }\mathrm{py}\left(1-e^{-\log R\cdot C/C_{\mathrm{ref}}}\right)dA$$

Finally, we estimate the cumulative impact of unit emissions from each source by integrating the effect across all receptors within the 72 km circle. This produces, in total, five values for each source on our 500 m grid: the marginal asthma prevalence due to an additional metric ton of NO_*x*_ emissions, and the marginal asthma rate change due to an additional metric ton of NO_*x*_ emissions for four race/ethnic groups (white non-Hispanic, Black, Asian, and Hispanic).

While MANE’s air quality modeling was performed on a regular 500 m grid spaced across the contiguous U.S., it is often more convenient to work with Census geographies, as emissions inventories are more commonly available at county-resolution. We converted the five calculated values for each source in our 500 m grid to the block group-level by taking the area-weighted average of the values across all sources on our grid that are located within each block group. We then aggregated up nested Census geographies (block groups, tracts, counties, and states) using a population-weighted average. The resulting values in larger Census geographies implicitly assume that NO_*x*_ emissions are correlated with population.

It is worth noting that the interpretation of the calculated sensitivities here differ from those in other reduced-complexity models. Most reduced-complexity models that predict the sensitivity of premature mortality to perturbations of emissions, the effect compounds each year; a reduction in emissions one year yields some reduction in premature mortality, and maintaining that emissions reduction year after year yields the same benefit each year. Here, however, we are looking at chronic disease prevalence, something that does not compound each year. A reduction in NO_*x*_ emissions one year will not cure children of asthma who already have it. Instead, a sustained reduction in emissions will reduce annual-average NO_2_ concentrations, which, in the long term, will prevent some children from developing asthma, reducing asthma prevalence over time.

### Model Evaluation

AERMOD has already been extensively evaluated against field campaigns,^30^ and so we do not directly evaluate the air quality model itself. Instead, we consider how uncertainty in concentration predictions from AERMOD, as well as other inputs into eq 1, impact the results from MANE.

To evaluate the uncertainty in our linear sensitivities of health end points to marginal changes in emissions, we use Monte Carlo analysis on the distribution of model inputs. The U.S. Census provides errors on population estimates, allowing us to sample the population from a distribution. The CDC provides lower and upper bounds on the baseline pediatric asthma rate for each individual Census tract, also allowing us to sample the asthma rate from a distribution.^29^ Epidemiological literature provides distributions on risk ratio, and evaluation studies of the PVMRM method allow us to empirically correct our concentration predictions as a function of distance from the source.

We perform Monte Carlo analysis, with specifics described in the next paragraph, for more than 500,000 source locations across the U.S. We sampled the source locations stratified by population density, with equal distribution across ten population deciles.

For a single Monte Carlo iteration at a single source location, we do the following. Details about the distributions are provided in Section S2.

1. For each Census tract within 72 km of the source, we sample the child population from a normal distribution *N*(μ, σ), where μ is the mean population estimate and σ is the margin of error divided by 1.96 (corresponding to the z-score for a 95% confidence interval), both from the 2020 Decennial Census.^28^
2. Also for each Census tract within 72 km of the source, we sample the baseline pediatric asthma rate from a uniform distribution defined by the upper and lower confidence limits from the PLACES data set.^29^
3. We sample the risk ratio from a uniform distribution from [1.03, 1.07] according to epidemiological literature.
4. We empirically correct the concentration prediction as a function of distance from the source based on evaluation studies of PVMRM, which show that the model potentially underestimates NO_2_ concentrations near the source (within ∼3 km) by up to 50% and overpredicts of NO_2_ concentrations further from the source by up to 50%.

With these parameters sampled, we then compute the marginal asthma prevalence as well as the marginal change in asthma rate by race/ethnicity using eq 1. For each source location, we repeat this Monte Carlo analysis 50 times, producing in total more than 25 million Monte Carlo simulations on which we performed our statistical analysis. Doing so produces 50 different estimates of marginal asthma prevalence and marginal change in asthma rate by race/ethnicity, allowing us to compute the median value and standard deviation in predicted metric. We define uncertainty as the relative spread of the predicted metric, σ/μ, which is computed separately for each of the 500,000 source locations in the sample.

To examine the contribution of each uncertain variable to the overall uncertainty in marginal asthma prevalence, we performed 50 Monte Carlo simulations for each source location under four different paradigms, with each paradigm corresponding to “turning off” the uncertainty of only one variable. For example, we performed Monte Carlo simulations where baseline pediatric asthma rate, risk ratio, and concentration were all sampled from their corresponding distributions, while assuming no uncertainty in population. The change in standard deviation of marginal asthma prevalence relative to the simulations where all uncertainties were considered indicates the relative contribution of the neglected variable to overall uncertainty. If the standard deviation does not change significantly, then the neglected variable does not significantly contribute to the overall uncertainty. Conversely, if the standard deviation noticeably decreases, then the neglected variable contributes significantly to the overall uncertainty.

### Emissions Inventories

We applied MANE to compare major source sectors of NO_*x*_, determining which source sectors contribute most significantly to overall asthma prevalence as well as which source sectors have disparate impacts on race/ethnic minorities. We identified five source sectors to analyze: on-road nondiesel light duty vehicles (LDV), on-road diesel heavy duty vehicles (HDV), commercial marine vessels (CMV), locomotives (LOC), and residential natural gas combustion (RNG). We used county-level NO_*x*_ emissions estimates for each source sector from the EPA’s 2020 National Emissions Inventory (NEI).^31^ These sectors collectively accounted for more than 3 million tons of NO_*x*_ emissions in 2020, approximately 45% of all nonpoint source emissions of NO_*x*_ and 34% of total NO_*x*_ emissions.

To apply MANE at the Census block group level, we need high spatial resolution inventories (i.e., at the Census block group level) for each of these source sectors. To do so, we downscaled county-level emissions from NEI using various spatial surrogates that we developed. Spatial surrogates are variables with a known spatial distribution that are used to approximate the spatial distribution of emissions; for a given surrogate variable representative of some source of emissions, we assume that the distribution of emissions within a county is perfectly correlated with the distribution of the surrogate variable within the same county. The spatial surrogates used for each source sector, along with the source of the data, are provided in Table 1. For CMV and RNG, the Census provides block group level estimates of the surrogate variable directly. For LDV, HDV, and LOC, we intersected the relevant geometries (road network, freight network, and rail network, respectively) with the Census block group geometry to generate block group-level estimates of the relevant variable.

**Table 1:** Spatial Surrogates Used to Develop High-Resolution Emissions Inventories for Five Source Sectors

source sector	spatial surrogate	surrogate source
nondiesel light-duty vehicles	lane-miles of road	openstreetmap^32^
diesel heavy-duty vehicles	ton-mileage of freight	federal highway administration^33^
commercial marine vessels	area of water	2020 decennial census^28^
locomotives	miles of rail	2020 decennial census^28^
residential natural gas combustion	households with natural gas heating	2018 american community survey^34^

To assess how uncertainty in distribution of emissions across an individual county affects the predicted asthma burden, we considered two other downscaling scenarios based on (1) population and (2) area. Downscaling emissions according to population will tend to produce a larger estimate of asthma burden, as emissions will occur in the more densely populated regions of the county, resulting in higher asthma burden. On the other hand, downscaling emissions according to area will tend to produce a lower estimate of asthma burden, since Census geographies are designed to have similar populations, and so downscaling emissions with area will put emissions in the less densely populated regions of the county, where the emissions will be less damaging. These two scenarios, coupled with the “best guess” estimate using an appropriate spatial surrogate, provide a range of estimated asthma burden for each source sector. While this is not a full treatment of the uncertainty associated with the spatial distribution of emissions, it provides reasonable bounds on predictions of asthma prevalence; rigorous validation of spatial surrogates should be a subject of future work.

## Results

### Model Results

Figure 2 shows the marginal asthma prevalence caused by an additional ton of NO_*x*_ emissions in each Census block group, one of the main results from the MANE model. On a national scale, the value of this metric varies by many orders of magnitude, with rural areas having very low values (<1 × 10^–4^ pediatric asthma cases per metric ton of NO_*x*_ emissions) and urban areas having more substantial values (on the order of 1 case per metric ton of NO_*x*_ emissions). The marginal asthma prevalence reaches its maximum value of approximately 14 cases of pediatric asthma caused per metric ton of NO_*x*_ emissions in New York City. There is an obvious and expected spatial trend, where emissions that occur in more densely populated areas result in higher asthma prevalence.

**Figure 2:**
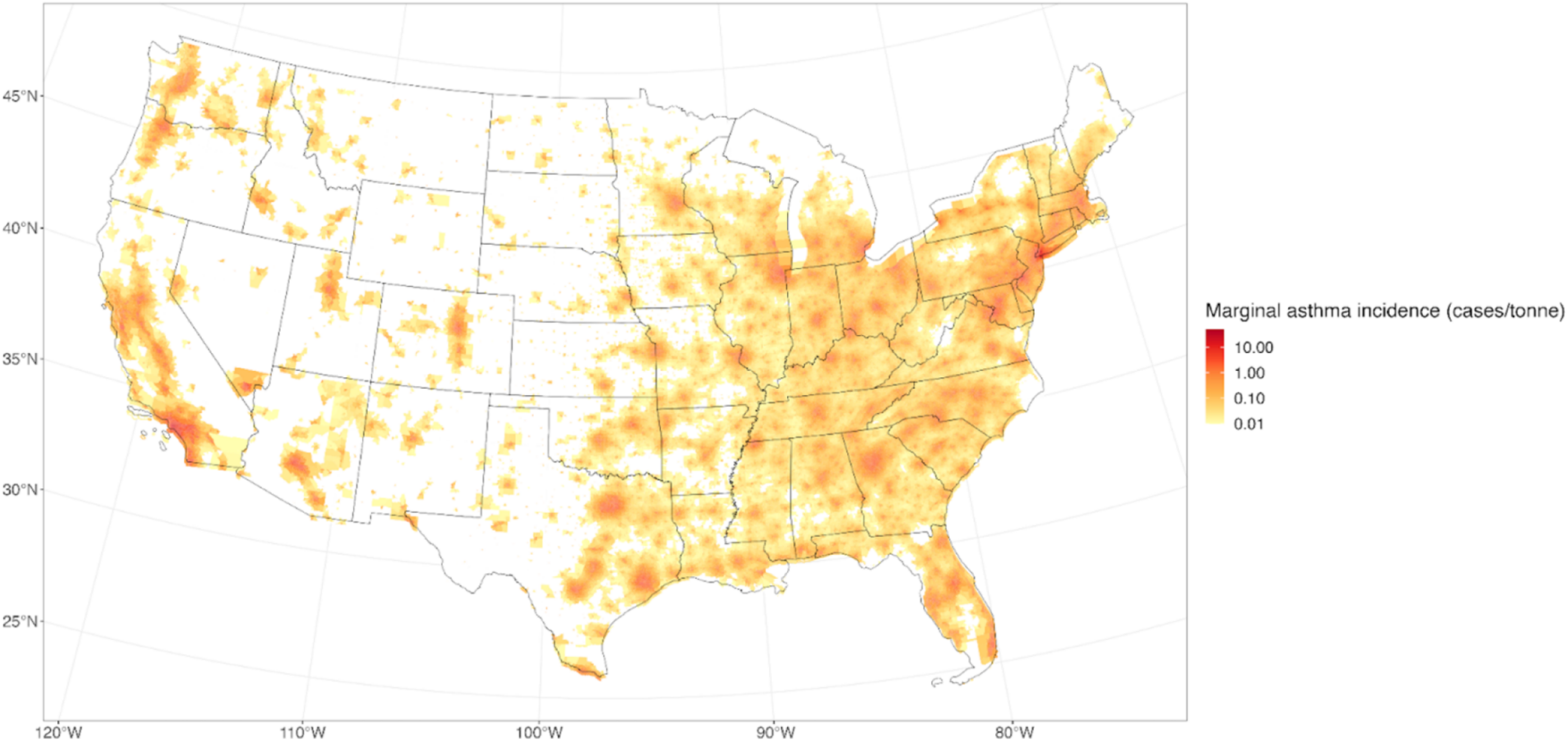
Marginal asthma prevalence per metric ton of NO_*x*_ emissions.

Figure 3 shows the marginal asthma prevalence caused by an additional metric ton of NO_*x*_ emissions in each Census block group across three metropolitan areas: New York, Los Angeles, and Chicago, respectively. Even within an urban area, this metric varies by multiple orders of magnitude, with emissions in the more suburban and rural areas of metropolitan areas having lower values than the urban core. This has implications for our analysis of specific source sectors; if emissions from some source sectors do not primarily occur in the most densely populated regions of a metropolitan area, then our county-level model, which assumes that emissions are correlated with population, will overestimate the asthma prevalence from those source sectors.

**Figure 3:**
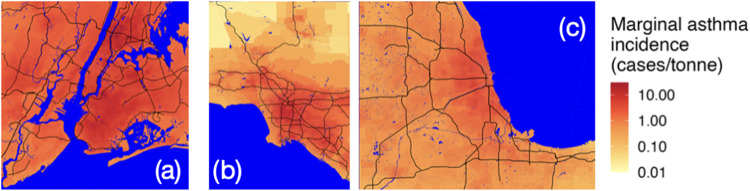
Marginal asthma prevalence by source location across (a) New York City, (b) Los Angeles, and (c) Chicago.

We also calculated the marginal change in asthma rate per capita caused by an additional metric ton of NO_*x*_ emissions in each Census block group for four race/ethnic groups: white non-Hispanic, Black, Asian, and Hispanic/Latino communities. Figure 4 shows the change in marginal asthma rate per capita in each race/ethnic group caused by an additional metric ton of NO_*x*_ emissions across New York, Los Angeles, and Chicago. Similar to total asthma prevalence (Figures 2 and 3), this metric varies by multiple orders of magnitude and displays marked spatial patterns reflecting the distribution of race/ethnicities across each metropolitan area, again emphasizing the importance of high spatial resolution. For example, in New York City, emissions in different boroughs affect different demographic groups; emissions in the Bronx disproportionately impact Black and Hispanic communities, while emissions in Queens have a larger impact on Asian and white non-Hispanic communities. Without this high spatial resolution, one would not be able to differentiate the relative impact of emissions in different parts of the same metropolitan area.

**Figure 4:**
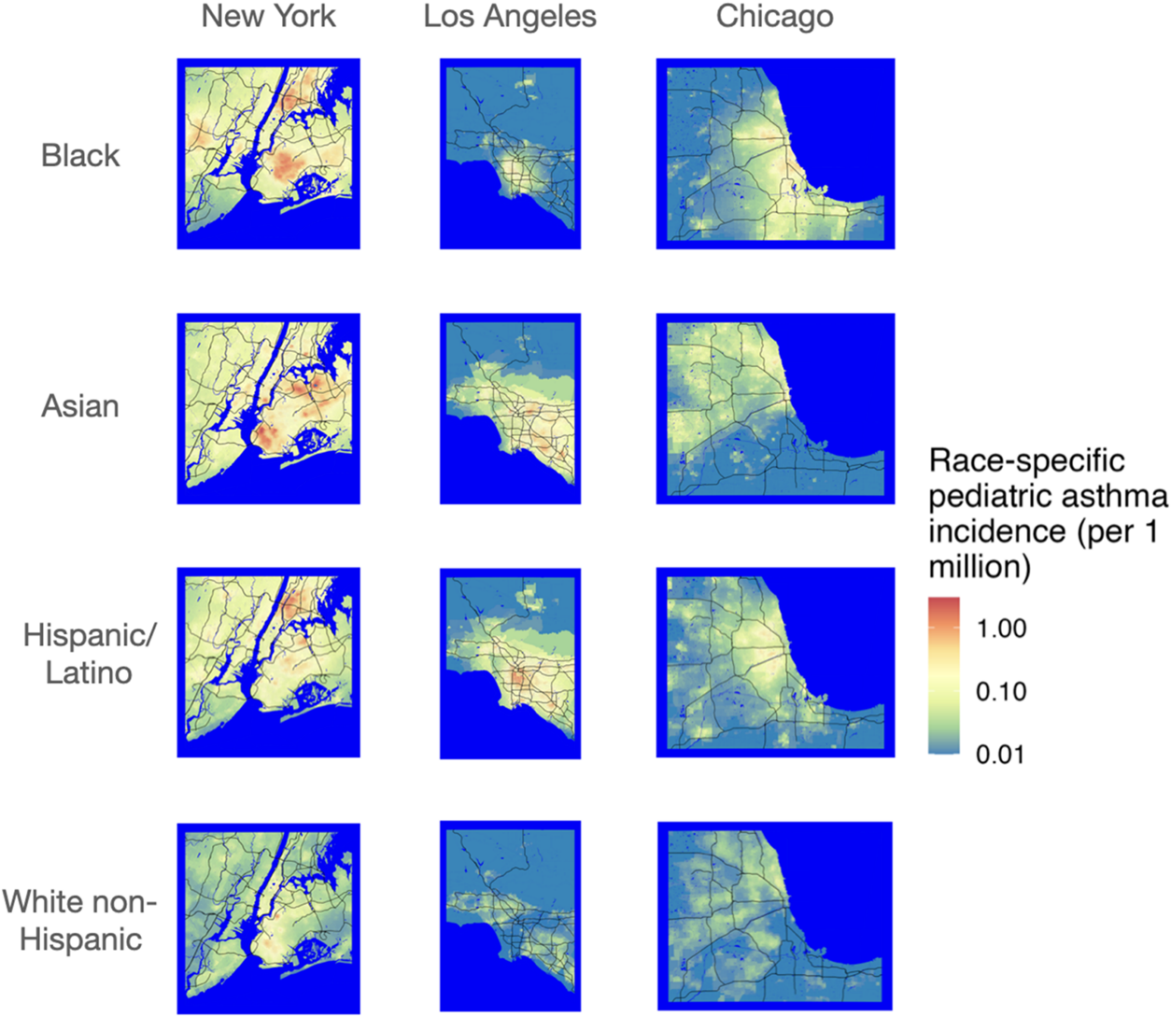
Marginal asthma prevalence per capita by race/ethnicity for source locations in three metropolitan areas: New York, Los Angeles, and Chicago.

### Model Evaluation and Uncertainty

Absent an analogous model to which we can compare, the primary evaluation of uncertainty of MANE relies on the uncertainty of our inputs into eq 1: population, baseline pediatric asthma rate, the risk ratio with its associated reference concentration, and the annual-average predicted NO_2_ concentrations. As described in the Methods section, we performed Monte Carlo simulations allowing the four inputs into the calculation to vary according to prescribed distributions.

Simulations where all four variables are allowed to vary according to their distributions allow us to directly quantify the resulting uncertainty in marginal asthma prevalence and prevalence by race as a result of uncertainty in inputs. For this analysis, we only looked at the asthma prevalence in Black children, but a similar pattern will hold for all race/ethnic results. We defined uncertainty as the relative spread of predicted marginal asthma prevalences for a single source location (σ/μ); these results are shown in Figure 5a,b for total marginal asthma prevalence and marginal asthma prevalence in Black children, respectively. Across all sources included in the Monte Carlo analysis, the median uncertainty value for predicted marginal asthma prevalence is 0.12, indicating that the relative size of the standard deviation in predicted marginal asthma prevalence is only 12% of the model’s best estimate. Additionally, the uncertainty decreases with population; for sources in the least populated regions of the U.S. (population decile 1), the median uncertainty is 17%, while in the most densely populated regions of the U.S. (population decile 10), the median uncertainty is 9.7%. The uncertainty in predicted asthma prevalence for only Black children is much larger; across all sources considered, the median uncertainty is 53%, with a significant difference in uncertainty between the lowest population deciles (120%) and the highest population deciles (15%). This indicates that for an individual source location, our predictions of total marginal asthma prevalence owing to emissions from that source carry with them an uncertainty of approximately 17%, while our predictions of marginal asthma prevalence by race/ethnicity may carry with them uncertainty above 50%.

**Figure 5:**
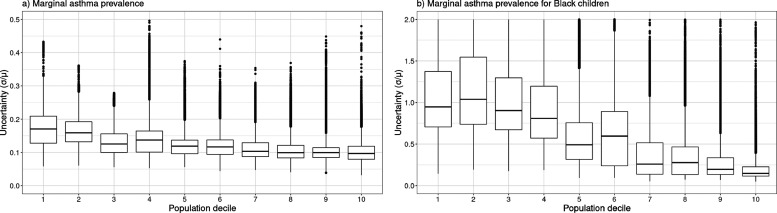
Modeled uncertainty in (a) total marginal asthma prevalence and (b) marginal asthma prevalence for Black children by population decile.

We also assessed the contribution of each variable individually to the overall uncertainty in total marginal asthma prevalence. To do so, we repeated the Monte Carlo simulations under four different paradigms, each of which omits the uncertainty of one variable. We tracked the percentage decrease in uncertainty as a result of omitting the variable, allowing us to assess the sensitivity of our modeled marginal asthma prevalence to uncertainty in the input variables.

Table 2 shows the percentage decrease in uncertainty of total marginal asthma prevalence as a result of omitting uncertainty in each individual variable. Uncertainty in risk ratio and concentration dominate the contributions to overall uncertainty; omitting the uncertainty in risk ratio reduces the uncertainty by almost 50%, and omitting uncertainty in concentration reduces uncertainty by almost 25%. On the other hand, uncertainty in population and baseline pediatric asthma rate contribute less to overall uncertainty, where omitting those uncertainties reduces overall uncertainty by only 0.3 and 1.7%, respectively. These percentage decreases do not sum to 100% due to interactions of uncertainty between the input variables not captured by the “leave-one-out” approach of our analysis. These changes in uncertainty make sense; population and baseline pediatric asthma rate have documented uncertainties that we treat as a random error at each individual receptor, i.e., the value at each receptor is unbiased with some uncertainty that can be either positive or negative. By treating this as random error, the cumulative error owing to uncertainty in population and baseline risk will be small when we sum across all receptors associated with a source (approximately 46,000 for each source), as the error in the sum of random variables decreases with the inverse square-root of the number of summands. On the other hand, the uncertainty associated with risk ratio and concentrations cannot be treated the same. The risk ratio is a global parameter that we assume does not vary with location, such that changing the risk ratio from the median estimate of 1.05 to the maximum estimate of 1.07 or the minimum estimate of 1.03 changes our estimate of asthma prevalence by 40%, a much larger source of uncertainty. Similarly, the empirical corrections we make to our concentration predictions are not random errors; evaluation studies of PVMRM demonstrate that it tends to underestimate maximum concentrations near the source, and overestimate the concentration further from the source. Accounting for this tendency of PVMRM to flatten out the concentration profile introduces significant uncertainty in model estimates, though not as much as the uncertainty in risk ratio.

**Table 2:** Percentage Decrease in Uncertainty of Total Marginal Asthma Prevalence as a Result of Omitting Uncertainty in Each Individual Variable

omitted uncertain variable	% decrease in uncertainty of total marginal asthma prevalence
population	0.3
baseline pediatric asthma rate	1.7
risk ratio	47.5
concentration	23.5

So far, our uncertainty analysis has focused on individual locations, i.e., the above work quantifies the uncertainty in predicted marginal asthma prevalence and prevalence by race/ethnicity owing to emissions from a single location. This work shows that the uncertainty for a single source can be significant. However, when this model is applied to analyze emissions from a large number of sources, where emissions are changing at many of the 40 million source locations we modeled across the U.S., the actual uncertainty in aggregate asthma prevalence owing to those emissions inventories is much lower. We can neglect uncertainty in population and baseline pediatric asthma rate, since these do not substantially contribute to uncertainty in predicted asthma prevalence. While uncertainty in concentration does significantly contribute to overall uncertainty in asthma prevalence at a single source location, its cumulative effect when analyzing changes in emissions across the U.S. is small. The air quality model used produces a pseudorandom error at each source location, such that we expect errors to be uncorrelated across large distances, i.e., in different parts of the country. On the other hand, the risk ratio is a global parameter across all source locations, such that modifying the risk ratio biases predicted asthma prevalence uniformly. As a result, when we assess national emissions inventories, where emissions are changing across enough locations that the uncertainty from concentration cancels out, the only remaining uncertainty results from uncertainty in risk ratio.

### Policy Application: Attributing Asthma to Source Sectors of Nitrogen Oxides

We applied MANE to estimate the number of pediatric asthma cases, as well as their distribution across race/ethnicity, attributable to five source sectors of NO_*x*_: nondiesel light-duty vehicles (LDV), diesel heavy-duty vehicles (HDV), commercial marine vessels (CMV), locomotives (LOC), and residential natural gas combustion (RNG). We applied the model at the Census block group-resolution, using the spatial surrogates developed to downscale county-level emissions estimates from the NEI to the block group-level.

The total number of asthma cases caused by each source sector is shown in Figure 6. HDV is responsible for the most pediatric asthma cases across the five sectors analyzed here, with approximately 167,000 ± 65,000 cases attributable to HDV, about 4 ± 1.5% of the total pediatric asthma burden in the U.S. This is followed by LDV at 140,000 ± 56,000 cases, RNG at 77,000 ± 30,000 cases, LOC at 49,000 ± 19,000 cases, and CMV at 27,000 ± 10,000 cases. In total, these account for approximately 460,000 ± 180,000 pediatric asthma cases, 10 ± 4% of all pediatric asthma cases in the U.S. This aligns well with best epidemiological estimates; it is estimated that about 20% of all pediatric asthma cases in the U.S. are attributable to NO_2_ exposure, and these source sectors (which account for 34% of all NO_*x*_ emissions) constitute approximately half of these cases.

**Figure 6:**
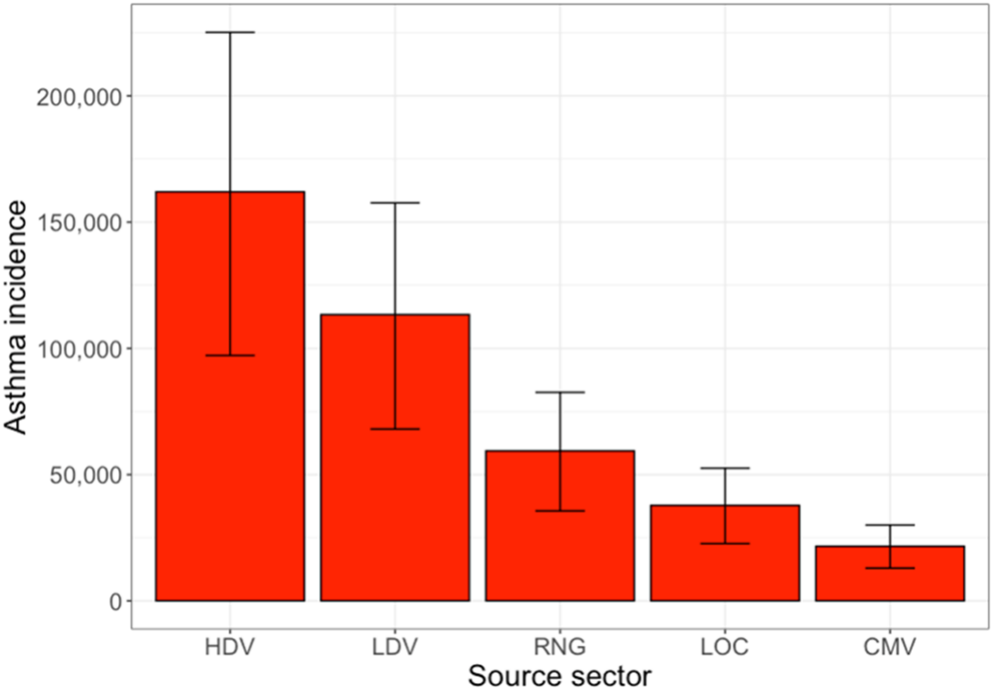
Predicted asthma prevalence by source sector of NO_*x*_. Error bars represent the 95% confidence interval on predicted asthma prevalence.

We also computed the predicted asthma prevalence using two alternate methods of downscaling emissions from the county-level to the block group-level: population downscaling and area downscaling. Our predicted asthma prevalence using all three downscaling methods are shown in Figure 7. As expected, using population to downscale emissions tends to produce a larger estimate of asthma prevalence, while using area to downscale emissions produces a lower estimate of asthma prevalence. There are two exceptions; using population to downscale RNG emissions produces a very similar asthma estimate as using the defined surrogate, and using area to downscale CMV emissions produces a larger estimate compared to the defined surrogate. These exceptions are sensible, as households with residential natural gas heating are likely to be highly correlated with population, yielding a similar asthma burden estimate, and emissions over water occur where there is not an immediate affected population, producing a lower asthma estimate. These results indicate that knowing the distribution of emissions within a county is important for accurate prediction of aggregate asthma prevalence; predictions of asthma prevalence can vary by up to 60% from the central estimate using an appropriate spatial surrogate. Previous work has indicated that high-resolution modeling is necessary for predicting exposure disparities;^19^ our work indicates that it is also important for an accurate prediction of aggregate public health impacts of NO_*x*_.

**Figure 7:**
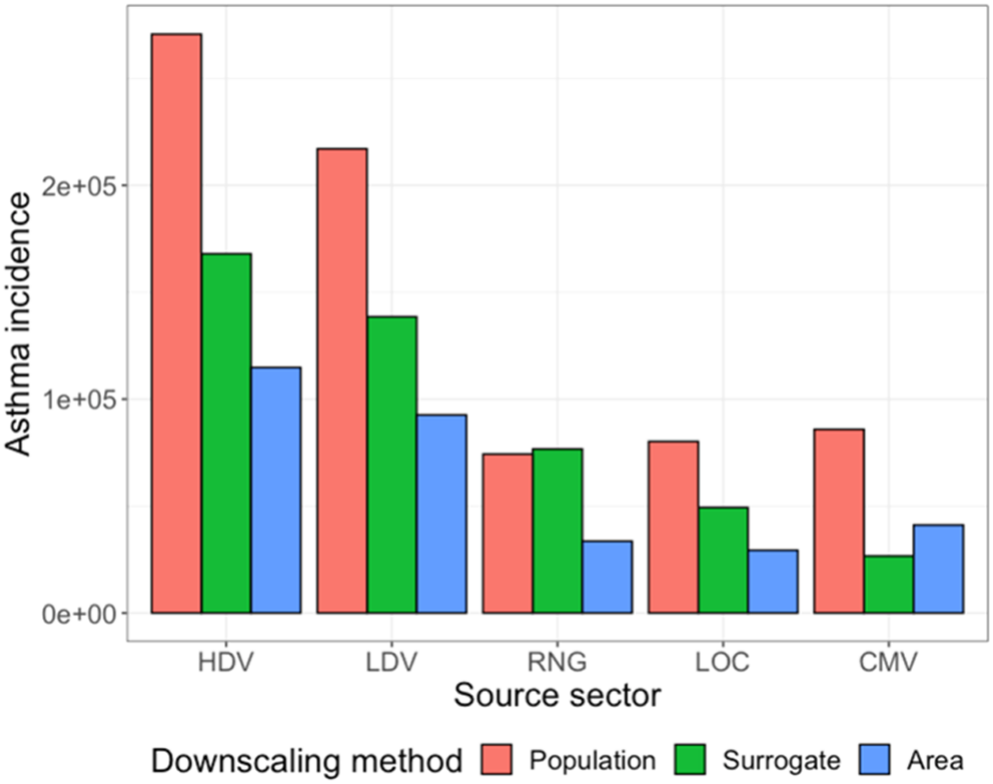
Predicted asthma prevalence for each source sector using different downscaling techniques. Error bars owing to uncertainty in risk ratio are omitted for clarity.

The asthma rate caused in four race/ethnic groups (white non-Hispanic, Black, Asian, and Hispanic) by each source sector is shown in Figure 8. The error bars represent the 95% confidence interval on asthma prevalence estimates due to uncertainty in risk ratio, a parameter that we assume is uniform across locations and race/ethnicity; as a result, the rank ordering of asthma prevalence by race/ethnicity is certain, even though the magnitude of asthma prevalence is uncertain. Each of these five source sectors disproportionately affects children of color; across all source sectors considered here, Black children experience the largest asthma burden, followed by Asian, Hispanic, and white non-Hispanic children. The asthma rate in Black children caused by each source sector is 65–100% higher than that in white non-Hispanic children, demonstrating consistent, systemic disproportionate impact of NO_*x*_ emissions on Black children.

**Figure 8:**
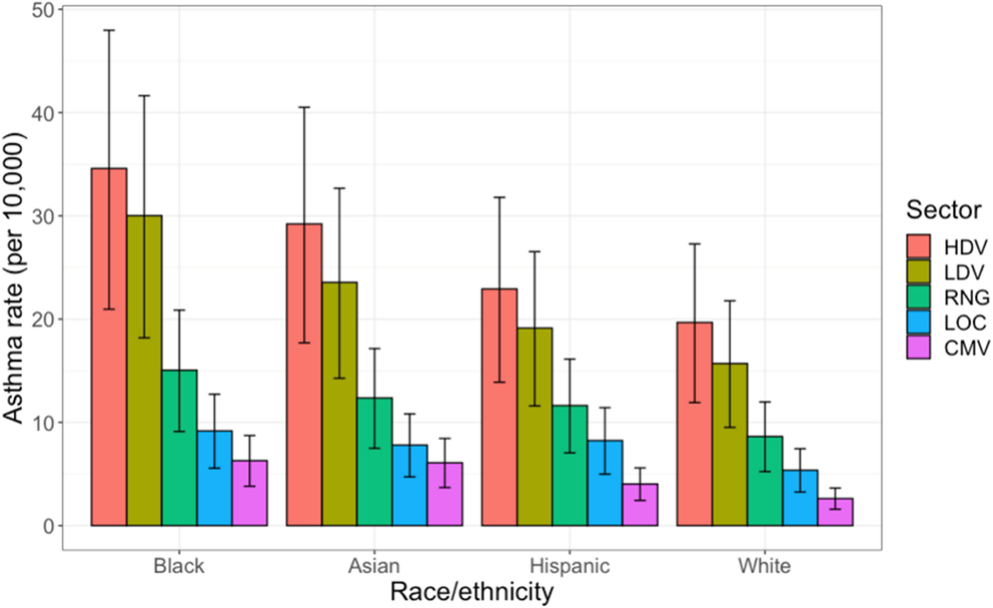
Predicted asthma prevalence by race/ethnicity for each source sector. Error bars represent the 95% confidence interval on predicted asthma prevalence by race/ethnicity.

Epidemiological literature suggests that the NO_2_-attributable percentage of pediatric asthma cases is higher in more densely populated areas. We disaggregated our total asthma prevalence results by metropolitan area, estimating the number of asthma cases caused by each source sector in 23 major metropolitan areas across the U.S. Table S1 shows these results. For all source sectors, emissions in the New York City metropolitan area cause the highest asthma prevalence, an expected result due to the high population density of New York. Light-duty vehicle traffic is responsible for a substantial number of asthma cases along the Northeast Corridor, with cities like Washington DC. Philadelphia, and Boston appearing near the top. Heavy-duty vehicle traffic is especially impactful in New York, with 33% of all asthma cases nationally attributable to heavy-duty vehicles occurring due to just traffic in New York. Commercial marine vessels, as expected, have higher emissions in major port cities, and therefore cities such as Houston and Seattle have a higher number of asthma cases attributable to commercial marine vessels. Similarly, major rail hubs such as Los Angeles and Chicago have higher locomotive emissions, and thus show peaks not seen in vehicular emissions. Residential natural gas combustion is responsible for more asthma cases in cooler-climate cities, an expected trend due to the use of natural gas for heat.

When we normalize the results in Table S1 by the associated magnitude of NO_*x*_ emissions, we can compare directly the relative impact of emissions across both metropolitan areas and source sectors. These results are shown in Table S2. Across all source sectors, emissions in New York and Los Angeles cause the most number of asthma cases per unit emission, reinforcing the importance of proximal population density to sources of NO_*x*_. The largest values are achieved by commercial marine vessels in both New York and Los Angeles, as well as locomotive emissions in Los Angeles and diesel heavy-duty vehicles in New York, suggesting that these are the source sectors that each metropolitan area should prioritize to maximize the reduction in asthma prevalence from reductions in NO_*x*_ emissions.

## Discussion

To our knowledge, MANE is the first model to directly predict pediatric asthma prevalence from NO_*x*_ emissions. Using a high-resolution Gaussian plume modeling framework, MANE provides block group-level estimates of the marginal asthma prevalence, as well as the asthma prevalence by race/ethnicity, owing to NO_*x*_ emissions from each individual block group. We applied MANE to estimate the pediatric asthma burden attributable to major sources of NO_*x*_, finding that heavy-duty vehicles are responsible for more than 4% of all pediatric asthma cases and 25% of pediatric asthma cases attributable to NO_2_ exposure. Additionally, across all source sectors we considered, the emissions cause 65–100% higher asthma prevalence in Black children compared to white non-Hispanic children, demonstrating consistent disproportionate impact. These impacts are also highly localized; for example, emissions from heavy-duty vehicles in the New York metropolitan area are responsible for more than 1.4% of all pediatric asthma cases nationally, suggesting that local, targeted policies restricting NO_*x*_ emissions may provide significant national public health benefit.

Our policy analysis focused only on the top five nonpoint sources of NO_*x*_ in the U.S., which constitute 50% of all nonpoint source NO_*x*_ emissions. We found that these source sectors cause a substantial number of pediatric asthma cases and that these cases disproportionately impact minority communities. However, there may be other source sectors that have lower overall emissions but higher asthma/environmental justice impacts due to the spatial distribution of those emissions. Future work should focus on quantifying pediatric asthma burden attributable to a wider variety of source sectors, with appropriate spatial surrogates developed for each sector analyzed.

One of the source sectors included in our policy application was residential natural gas combustion (RNG). Natural gas is combusted in residential buildings for both heating and cooking, and cooking emissions are likely to produce higher indoor NO_2_ concentrations that children in the home are exposed to. Our estimate of marginal asthma prevalence from outdoor NO_2_ concentrations from RNG is therefore likely an underestimate of the total number of pediatric asthma cases attributable to RNG, something that can be explored in future work.

Our modeling framework to predict marginal asthma prevalence from emissions of NO_*x*_ has previously been applied to predict premature mortality from emissions of primary PM_2.5_; this same modeling framework is broadly applicable for predicting disease or mortality prevalence from perturbations in emissions of any air pollutant, provided a relationship between disease or mortality prevalence and air pollutant concentrations exists. Future work in the development of reduced-complexity air quality models could apply this same modeling framework to other diseases or air pollutants of import.

MANE satisfies an important niche in a policy context, allowing policymakers to rapidly assess the cumulative public health benefits of policies restricting emissions of NO_*x*_. MANE also has an important environmental justice focus, allowing one to address equity concerns by assessing the distribution of asthma prevalence across race/ethnicity. Both of these factors make MANE a key screening tool for potential policies affecting asthma prevalence.

## Supplementary Material



## Notes

Map of meteorological regions used in MANE; pediatric asthma cases by metropolitan area; discussion of sensitivity analysis (PDF)
