# Supplementary material for: Marginal Asthma Prevalence from NO x Emissions (MANE): A Model to Predict Pediatric Asthma Burden from Emissions of Nitrogen Oxides
Source: Environ Sci Technol. 2025 May 19;59(21):10347–56. doi: 10.1021/acs.est.4c09012 (PMC12139029; doi:10.1021/acs.est.4c09012)
Supplement: Supplementary file 1 [file es4c09012_si_001.pdf]

Supporting Information for Marginal Asthma prevalence from NO_x Emissions (MANE): A model to predict pediatric asthma burden from emissions of nitrogen oxides

Brian M. Gentry[1*], Allen L. Robinson[2], Peter J. Adams[1]
[1]Department of Engineering and Public Policy, Carnegie Mellon University, 5000 Forbes Ave., Pittsburgh, PA 15213
[2]Department of Atmospheric Sciences, Colorado State University, 200 W. Lake St., Fort Collins, CO 80521

*email: bgentry@alumni.cmu.edu

Summary: 19 pages, 1 figure, 2 tables, 3 sections

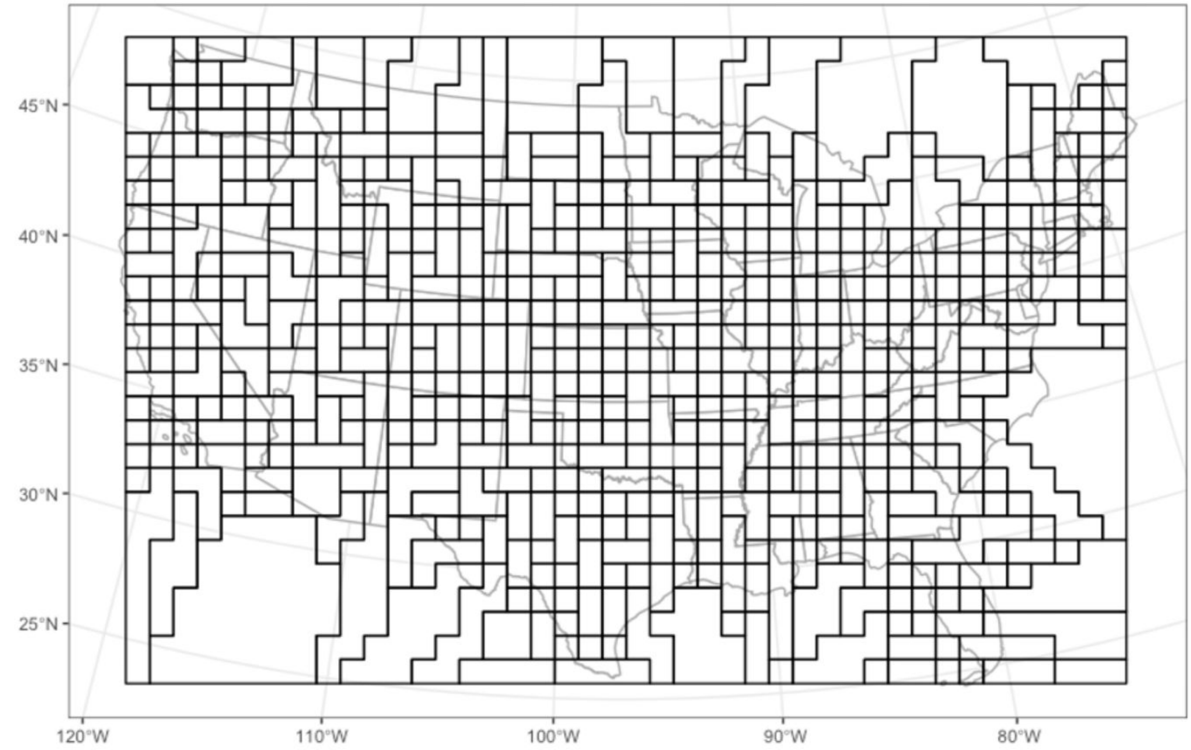

Figure S1: Map of meteorology regions (black grid cells) covering the contiguous U.S.

Table S1: Predicted asthma prevalence for each source sector by metropolitan area (total cases).

Metro area	HDV	LDV	RNG	LOC	CMV
**New York-Newark, NY-NJ-CT-PA**	62289	22953	17574	7970	26429
**Philadelphia-Reading-Camden, PA-NJ-DE-MD**	8329	5742	8101	2265	4811
**Washington-Baltimore-Arlington, DC-MD-VA-WV-PA**	8110	9668	7585	2067	2171
**Atlanta--Athens-Clarke County--Sandy Springs, GA-AL**	5952	5192	2913	1273	0
**Boston-Worcester-Providence, MA-RI-NH-CT**	4967	4416	4808	1771	2152
**San Jose-San Francisco-Oakland, CA**	3602	3383	3102	2445	1589
**Portland-Vancouver-Salem, OR-WA**	3558	2058	1629	936	1607
**Denver-Aurora, CO**	3222	3373	2904	505	0
**Minneapolis-St. Paul, MN-WI**	3203	2192	1341	808	91
**Los Angeles-Long Beach, CA**	3074	4412	3240	4498	7600

**Detroit-Warren-Ann Arbor, MI**	3069	1644	678	599	296
**Chicago-Naperville, IL-IN-WI**	3011	3327	1747	2826	1088
**Dallas-Fort Worth, TX-OK**	2557	2095	1433	1152	0
**Cincinnati-Wilmington-Maysville, OH-KY-IN**	2525	2358	1449	654	173
**St. Louis-St. Charles-Farmington, MO-IL**	2404	2458	886	605	901
**Indianapolis-Carmel-Muncie, IN**	2195	1322	1005	367	0
**Cleveland-Akron-Canton, OH**	1760	2299	1134	1345	367
**Orlando-Lakeland-Deltona, FL**	1703	1896	100	207	9
**Kansas City-Overland Park-Kansas City, MO-KS**	1684	1411	1015	857	12
**Houston-The Woodlands, TX**	1664	1132	392	895	3977
**New Orleans-Metairie-Hammond, LA-MS**	1569	794	245	170	3352
**Salt Lake City-Provo-Orem, UT**	1501	1770	547	445	0
**Sacramento-Roseville, CA**	1464	1239	868	453	33
**Seattle-Tacoma, WA**	1247	973	593	706	2528
**Nashville-Davidson--Murfreesboro, TN**	1238	1103	332	0	22
**Columbus-Marion-Zanesville, OH**	1159	637	652	579	0
**Louisville/Jefferson County--Elizabethtown--Bardstown, KY-IN**	1145	780	572	207	175
**Harrisburg-York-Lebanon, PA**	1134	702	570	525	0
**Albany-Schenectady, NY**	1110	174	453	165	49
**Milwaukee-Racine-Waukesha, WI**	1062	1095	361	344	198
**Charlotte-Concord, NC-SC**	1061	1703	651	308	0
**Greenville-Spartanburg-Anderson, SC**	984	848	147	121	0
**Miami-Port St. Lucie-Fort Lauderdale, FL**	887	857	135	366	3193
**Columbia-Orangeburg-Newberry, SC**	846	568	105	65	0
**San Antonio-New Braunfels-Pearsall, TX**	812	711	526	0	0
**Jacksonville-St. Marys-Palatka, FL-GA**	810	432	24	150	1298
**Greensboro--Winston-Salem--High Point, NC**	787	832	194	0	0
**Syracuse-Auburn, NY**	764	153	469	210	13
**Hartford-East Hartford, CT**	760	937	1077	216	25
**Oklahoma City-Shawnee, OK**	755	473	451	154	0

**Toledo-Findlay-Tiffin, OH**	731	454	528	546	122
**Rochester-Batavia-Seneca Falls, NY**	728	283	76	262	39
**Virginia Beach-Norfolk, VA-NC**	703	1562	445	151	3909
**Knoxville-Morristown-Sevierville, TN**	685	432	132	133	1
**Birmingham-Hoover-Talladega, AL**	674	349	182	381	4
**Grand Rapids-Kentwood-Muskegon, MI**	655	705	757	53	62
**Memphis-Forrest City, TN-MS-AR**	636	668	70	415	487
**Pittsburgh-New Castle-Weirton, PA-OH-WV**	604	502	433	773	155
**Lexington-Fayette--Richmond--Frankfort, KY**	603	394	131	139	0
**Raleigh-Durham-Cary, NC**	588	981	333	0	0
**Phoenix-Mesa, AZ**	562	191	863	463	0
**Omaha-Council Bluffs-Fremont, NE-IA**	561	813	471	274	0
**Little Rock-North Little Rock, AR**	520	391	134	228	81
**Boise City-Mountain Home-Ontario, ID-OR**	517	579	444	135	0
**Pensacola-Ferry Pass, FL-AL**	514	237	24	27	193
**Chattanooga-Cleveland-Dalton, TN-GA**	512	491	74	264	42
**Mobile-Daphne-Fairhope, AL**	497	331	82	78	775
**Fresno-Madera-Hanford, CA**	495	527	710	923	0
**Buffalo-Cheektowaga-Olean, NY**	452	289	114	300	51
**Des Moines-Ames-West Des Moines, IA**	428	296	238	103	0
**Columbus-Auburn-Opelika, GA-AL**	410	267	70	64	0
**Fort Wayne-Huntington-Auburn, IN**	405	301	218	214	0
**Dayton-Springfield-Kettering, OH**	400	409	309	161	0
**Cape Coral-Fort Myers-Naples, FL**	396	305	4	7	11
**Macon-Bibb County--Warner Robins, GA**	387	340	49	84	0
**North Port-Sarasota, FL**	382	303	20	11	156
**Wichita-Winfield, KS**	376	284	222	170	0
**Shreveport-Bossier City-Minden, LA**	369	210	59	141	1
**Tulsa-Muskogee-Bartlesville, OK**	351	663	415	207	6
**Tucson-Nogales, AZ**	337	239	250	197	0
**Lafayette-Opelousas-Morgan City, LA**	336	193	101	126	239

**Charleston-Huntington-Ashland, WV-OH-KY**	317	161	115	218	96
**Albuquerque-Santa Fe-Las Vegas, NM**	315	342	509	0	0
**Las Vegas-Henderson, NV**	312	140	1	157	0
**Spokane-Spokane Valley-Coeur d'Alene, WA-ID**	295	163	279	112	0
**Johnson City-Kingsport-Bristol, TN-VA**	285	241	21	46	0
**Montgomery-Selma-Alexander City, AL**	285	305	95	148	0
**South Bend-Elkhart-Mishawaka, IN-MI**	267	383	483	504	6
**Amarillo-Pampa-Borger, TX**	263	80	115	152	0
**Huntsville-Decatur, AL**	251	357	35	86	12
**Madison-Janesville-Beloit, WI**	251	186	240	35	0
**Jackson-Vicksburg-Brookhaven, MS**	249	268	84	118	31
**Rockford-Freeport-Rochelle, IL**	245	189	270	37	0
**McAllen-Edinburg, TX**	241	76	11	13	1
**Youngstown-Warren, OH-PA**	231	182	241	183	3
**Midland-Odessa, TX**	226	101	58	26	0
**Lubbock-Plainview-Levelland, TX**	224	127	97	23	0
**Fayetteville-Sanford-Lumberton, NC**	199	457	93	97	0
**Appleton-Oshkosh-Neenah, WI**	197	109	203	63	1
**Reno-Carson City-Fernley, NV**	194	425	304	39	0
**Bloomsburg-Berwick-Sunbury, PA**	191	104	56	19	0
**Lake Charles-Jennings, LA**	189	107	30	56	430
**Cedar Rapids-Iowa City, IA**	181	102	120	73	0
**Idaho Falls-Rexburg-Blackfoot, ID**	180	60	62	9	0
**Lincoln-Beatrice, NE**	172	101	120	168	0
**Gainesville-Lake City, FL**	164	78	11	35	0
**Kalamazoo-Battle Creek-Portage, MI**	164	157	173	39	1
**Savannah-Hinesville-Statesboro, GA**	158	260	48	86	805
**Portland-Lewiston-South Portland, ME**	153	85	45	22	123
**Kennewick-Richland-Walla Walla, WA**	150	169	55	76	38
**Davenport-Moline, IA-IL**	148	126	178	86	62
**Green Bay-Shawano, WI**	139	106	120	15	27

**Corpus Christi-Kingsville-Alice, TX**	139	137	31	16	1266
**Harrisonburg-Staunton, VA**	133	79	14	21	0
**Salisbury-Cambridge, MD-DE**	129	101	48	12	195
**Asheville-Marion-Brevard, NC**	116	118	18	28	0
**Springfield-Jacksonville-Lincoln, IL**	114	74	150	70	5
**Saginaw-Midland-Bay City, MI**	113	91	136	18	6
**Myrtle Beach-Conway, SC-NC**	111	101	13	25	42
**Joplin-Miami, MO-OK**	106	52	28	56	0
**Bowling Green-Glasgow, KY**	105	46	27	23	0
**El Paso-Las Cruces, TX-NM**	105	124	281	151	0
**Pueblo-Cañon City, CO**	100	94	470	116	0
**Eau Claire-Menomonie, WI**	100	26	29	12	0
**Tyler-Jacksonville, TX**	98	66	29	35	0
**Columbia-Moberly-Mexico, MO**	95	58	34	25	1
**Monroe-Ruston, LA**	95	65	17	53	4
**Brownsville-Harlingen-Raymondville, TX**	94	63	7	12	42
**Lafayette-West Lafayette-Frankfort, IN**	93	54	63	60	0
**Redding-Red Bluff, CA**	92	23	29	24	0
**Fargo-Wahpeton, ND-MN**	89	25	50	60	0
**Jackson-Brownsville, TN**	89	65	24	27	6
**Lima-Van Wert-Celina, OH**	86	48	36	43	0
**Victoria-Port Lavaca, TX**	84	11	6	0	52
**State College-DuBois, PA**	83	23	21	9	0
**Wausau-Stevens Point-Wisconsin Rapids, WI**	83	34	41	44	0
**Erie-Meadville, PA**	81	118	144	90	32
**Mansfield-Ashland-Bucyrus, OH**	74	45	42	98	0
**Medford-Grants Pass, OR**	74	55	21	4	0
**Hattiesburg-Laurel, MS**	73	45	10	30	0
**Bloomington-Bedford, IN**	71	45	38	10	0
**Bloomington-Pontiac, IL**	65	33	88	51	6
**Rocky Mount-Wilson-Roanoke Rapids, NC**	64	99	12	39	0
**Tupelo-Corinth, MS**	58	36	8	24	1

**Ithaca-Cortland, NY**	58	11	99	30	0
**Morgantown-Fairmont, WV**	56	25	30	22	5
**Elmira-Corning, NY**	52	8	58	21	0
**Dothan-Ozark, AL**	47	64	3	7	0
**Paducah-Mayfield, KY-IL**	47	26	13	22	101
**Jonesboro-Paragould, AR**	44	33	14	75	0
**Edwards-Glenwood Springs, CO**	43	20	69	11	0
**Parkersburg-Marietta-Vienna, WV-OH**	42	26	28	2	25
**Rochester-Austin, MN**	41	42	43	21	2
**Greenville-Kinston-Washington, NC**	40	82	12	8	1
**Cape Girardeau-Sikeston, MO-IL**	35	23	17	38	21
**Scottsboro-Fort Payne, AL**	34	38	2	33	1
**Altoona-Huntingdon, PA**	33	53	47	80	0
**Johnstown-Somerset, PA**	30	35	27	65	0
**Dixon-Sterling, IL**	25	11	23	62	2
**Hot Springs-Malvern, AR**	23	13	5	7	0
**Bend-Prineville, OR**	23	31	12	7	0
**Rapid City-Spearfish, SD**	22	22	21	4	0
**New Bern-Morehead City, NC**	20	53	11	5	12
**Quincy-Hannibal, IL-MO**	20	14	25	19	19
**Columbus-West Point, MS**	18	12	4	8	2
**Mankato-New Ulm, MN**	18	12	20	5	0
**Moses Lake-Othello, WA**	18	19	2	14	0
**Pullman-Moscow, WA-ID**	17	10	11	9	3
**Burlington-South Burlington-Barre, VT**	16	19	1	9	0
**Williamsport-Lock Haven, PA**	15	15	32	9	0
**Mount Pleasant-Alma, MI**	15	16	16	2	0
**Richmond-Connersville, IN**	14	10	14	25	0
**Kokomo-Peru, IN**	13	13	15	20	0
**Burlington-Fort Madison-Keokuk, IA-IL-MO**	12	14	14	47	23
**Clovis-Portales, NM**	10	7	8	47	0
**DeRidder-Fort Polk South, LA**	9	6	1	11	0

**Cleveland-Indianola, MS**	9	5	7	5	24
**Martin-Union City, TN**	9	8	2	11	10
**Marinette-Iron Mountain, WI-MI**	8	5	11	5	4
**Kerrville-Fredericksburg, TX**	7	3	1	0	0
**Steamboat Springs-Craig, CO**	7	5	12	1	0
**Spencer-Spirit Lake, IA**	3	2	1	0	0

Table S2: Predicted asthma prevalence for each source sector by metropolitan area normalized by total sector emissions in that metropolitan area (cases/metric ton).

Metro area	HDV	LDV	RNG	LOC	CMV
**New York-Newark, NY-NJ-CT-PA**	1.67	1.16	1.13	1.06	2.64
**Los Angeles-Long Beach, CA**	1.39	0.97	1.20	1.50	2.81
**Philadelphia-Reading-Camden, PA-NJ-DE-MD**	0.61	0.60	0.84	0.63	1.03
**Washington-Baltimore-Arlington, DC-MD-VA-WV-PA**	0.42	0.57	1.01	0.31	0.75
**Boston-Worcester-Providence, MA-RI-NH-CT**	0.40	0.39	0.81	0.59	0.79
**Portland-Vancouver-Salem, OR-WA**	0.39	0.25	0.86	0.21	0.60
**Detroit-Warren-Ann Arbor, MI**	0.37	0.30	0.25	0.37	0.42
**Sacramento-Roseville, CA**	0.31	0.37	0.63	0.18	0.64
**Atlanta--Athens-Clarke County--Sandy Springs, GA-AL**	0.31	0.29	0.56	0.21	0.00
**San Jose-San Francisco-Oakland, CA**	0.29	0.32	0.50	0.37	0.26
**Hartford-East Hartford, CT**	0.28	0.33	0.65	0.33	0.15
**Cincinnati-Wilmington-Maysville, OH-KY-IN**	0.28	0.32	0.52	0.26	0.39
**Minneapolis-St. Paul, MN-WI**	0.28	0.28	0.36	0.16	0.58
**Denver-Aurora, CO**	0.26	0.36	0.58	0.14	0.01
**St. Louis-St. Charles-Farmington, MO-IL**	0.24	0.31	0.39	0.10	0.47
**Harrisburg-York-Lebanon, PA**	0.23	0.19	0.56	0.24	0.31
**Chicago-Naperville, IL-IN-WI**	0.22	0.35	0.33	0.27	0.86
**Virginia Beach-Norfolk, VA-NC**	0.22	0.30	0.49	0.12	0.55

**Grand Rapids-Kentwood-Muskegon, MI**	0.21	0.19	0.36	0.16	0.19
**Buffalo-Cheektowaga-Olean, NY**	0.21	0.23	0.21	0.20	0.24
**New Orleans-Metairie-Hammond, LA-MS**	0.21	0.19	0.33	0.10	0.31
**Phoenix-Mesa, AZ**	0.21	0.09	0.90	0.21	0.39
**Seattle-Tacoma, WA**	0.21	0.14	0.40	0.18	0.33
**Milwaukee-Racine-Waukesha, WI**	0.20	0.32	0.22	0.19	0.70
**Indianapolis-Carmel-Muncie, IN**	0.20	0.18	0.37	0.15	0.00
**Miami-Port St. Lucie-Fort Lauderdale, FL**	0.19	0.26	0.46	0.30	0.67
**Cleveland-Akron-Canton, OH**	0.18	0.32	0.30	0.21	0.37
**Pensacola-Ferry Pass, FL-AL**	0.18	0.12	0.33	0.05	0.37
**Las Vegas-Henderson, NV**	0.18	0.13	0.04	0.12	0.99
**Salt Lake City-Provo-Orem, UT**	0.17	0.26	0.33	0.08	0.07
**Charlotte-Concord, NC-SC**	0.17	0.16	0.37	0.20	0.00
**Greensboro--Winston-Salem--High Point, NC**	0.17	0.13	0.30	0.00	0.00
**Raleigh-Durham-Cary, NC**	0.17	0.16	0.36	0.00	0.00
**Mobile-Daphne-Fairhope, AL**	0.17	0.15	0.42	0.08	0.36
**Louisville/Jefferson County--Elizabethtown--Bardstown, KY-IN**	0.17	0.20	0.53	0.20	0.27
**McAllen-Edinburg, TX**	0.16	0.15	0.55	0.14	0.01
**Tucson-Nogales, AZ**	0.16	0.12	0.46	0.12	0.03
**Dallas-Fort Worth, TX-OK**	0.16	0.29	0.59	0.20	0.00
**Toledo-Findlay-Tiffin, OH**	0.16	0.17	0.32	0.11	0.34

**Kansas City-Overland Park-Kansas City, MO-KS**	0.15	0.19	0.39	0.09	0.25
**Orlando-Lakeland-Deltona, FL**	0.15	0.24	0.34	0.15	0.11
**Syracuse-Auburn, NY**	0.15	0.17	0.41	0.13	0.08
**Greenville-Spartanburg-Anderson, SC**	0.15	0.12	0.22	0.11	0.00
**San Antonio-New Braunfels-Pearsall, TX**	0.15	0.23	0.60	0.00	0.00
**Columbus-Auburn-Opelika, GA-AL**	0.14	0.10	0.33	0.04	0.00
**Nashville-Davidson--Murfreesboro, TN**	0.14	0.18	0.29	0.00	0.25
**Fresno-Madera-Hanford, CA**	0.14	0.26	0.94	0.35	0.46
**Shreveport-Bossier City-Minden, LA**	0.14	0.12	0.28	0.11	0.29
**Jacksonville-St. Marys-Palatka, FL-GA**	0.13	0.14	0.24	0.08	0.38
**Houston-The Woodlands, TX**	0.13	0.25	0.44	0.19	0.58
**Memphis-Forrest City, TN-MS-AR**	0.13	0.25	0.19	0.13	0.43
**Columbus-Marion-Zanesville, OH**	0.13	0.12	0.27	0.17	0.05
**Columbia-Orangeburg-Newberry, SC**	0.13	0.12	0.24	0.09	0.00
**Rochester-Batavia-Seneca Falls, NY**	0.13	0.21	0.13	0.14	0.17
**Albany-Schenectady, NY**	0.13	0.12	0.38	0.10	0.36
**Spokane-Spokane Valley-Coeur d'Alene, WA-ID**	0.12	0.08	0.46	0.06	0.00
**Lexington-Fayette--Richmond--Frankfort, KY**	0.12	0.15	0.35	0.11	0.01
**Rockford-Freeport-Rochelle, IL**	0.12	0.15	0.31	0.06	0.01
**Omaha-Council Bluffs-Fremont, NE-IA**	0.12	0.23	0.45	0.08	0.02
**Boise City-Mountain Home-Ontario, ID-OR**	0.12	0.18	0.53	0.08	0.00

**Knoxville-Morristown-Sevierville, TN**	0.12	0.10	0.25	0.07	0.19
**Dayton-Springfield-Kettering, OH**	0.12	0.13	0.32	0.11	0.13
**Appleton-Oshkosh-Neenah, WI**	0.11	0.08	0.28	0.15	0.08
**Macon-Bibb County--Warner Robins, GA**	0.11	0.13	0.26	0.09	0.00
**Oklahoma City-Shawnee, OK**	0.11	0.13	0.36	0.08	0.00
**Lafayette-Opelousas-Morgan City, LA**	0.11	0.10	0.29	0.09	0.11
**Birmingham-Hoover-Talladega, AL**	0.11	0.07	0.27	0.11	0.08
**North Port-Sarasota, FL**	0.10	0.14	0.25	0.08	0.26
**Johnson City-Kingsport-Bristol, TN-VA**	0.10	0.10	0.18	0.08	0.00
**Cape Coral-Fort Myers-Naples, FL**	0.10	0.14	0.14	0.04	0.16
**Wichita-Winfield, KS**	0.10	0.12	0.32	0.04	0.04
**Montgomery-Selma-Alexander City, AL**	0.10	0.09	0.28	0.07	0.00
**Kennewick-Richland-Walla Walla, WA**	0.10	0.10	0.57	0.05	0.54
**Huntsville-Decatur, AL**	0.10	0.11	0.20	0.09	0.14
**Fort Wayne-Huntington-Auburn, IN**	0.10	0.11	0.27	0.08	0.06
**Little Rock-North Little Rock, AR**	0.10	0.12	0.22	0.07	0.22
**Fayetteville-Sanford-Lumberton, NC**	0.10	0.11	0.29	0.10	0.00
**Kalamazoo-Battle Creek-Portage, MI**	0.09	0.10	0.22	0.14	0.09
**Pittsburgh-New Castle-Weirton, PA-OH-WV**	0.09	0.09	0.18	0.16	0.25
**Youngstown-Warren, OH-PA**	0.09	0.10	0.22	0.12	0.04
**South Bend-Elkhart-Mishawaka, IN-MI**	0.09	0.14	0.26	0.14	0.13

**Saginaw-Midland-Bay City, MI**	0.09	0.09	0.22	0.13	0.13
**Harrisonburg-Staunton, VA**	0.09	0.07	0.22	0.06	0.00
**Green Bay-Shawano, WI**	0.09	0.08	0.22	0.06	0.07
**Des Moines-Ames-West Des Moines, IA**	0.09	0.10	0.26	0.04	0.00
**Tulsa-Muskogee-Bartlesville, OK**	0.09	0.18	0.37	0.07	0.17
**Bloomsburg-Berwick-Sunbury, PA**	0.09	0.08	0.35	0.12	0.00
**Monroe-Ruston, LA**	0.09	0.07	0.15	0.07	0.12
**Chattanooga-Cleveland-Dalton, TN-GA**	0.08	0.10	0.16	0.09	0.24
**Lincoln-Beatrice, NE**	0.08	0.07	0.34	0.06	0.00
**Lake Charles-Jennings, LA**	0.08	0.09	0.27	0.06	0.13
**Reno-Carson City-Fernley, NV**	0.08	0.14	0.44	0.03	0.00
**Brownsville-Harlingen-Raymondville, TX**	0.08	0.11	0.35	0.13	0.17
**Charleston-Huntington-Ashland, WV-OH-KY**	0.08	0.06	0.16	0.08	0.11
**Cedar Rapids-Iowa City, IA**	0.08	0.06	0.19	0.06	0.02
**Portland-Lewiston-South Portland, ME**	0.08	0.08	0.31	0.09	0.17
**Lubbock-Plainview-Levelland, TX**	0.08	0.11	0.29	0.04	0.00
**Pueblo-Cañon City, CO**	0.07	0.07	0.46	0.10	0.00
**Salisbury-Cambridge, MD-DE**	0.07	0.06	0.18	0.11	0.08
**Asheville-Marion-Brevard, NC**	0.07	0.05	0.13	0.06	0.00
**Jackson-Vicksburg-Brookhaven, MS**	0.07	0.09	0.22	0.07	0.05
**Lima-Van Wert-Celina, OH**	0.07	0.04	0.16	0.05	0.08

**Dothan-Ozark, AL**	0.07	0.05	0.12	0.05	0.01
**Midland-Odessa, TX**	0.06	0.11	0.34	0.09	0.00
**Idaho Falls-Rexburg-Blackfoot, ID**	0.06	0.03	0.34	0.02	0.00
**Erie-Meadville, PA**	0.06	0.11	0.33	0.11	0.32
**Madison-Janesville-Beloit, WI**	0.06	0.08	0.21	0.04	0.01
**Savannah-Hinesville-Statesboro, GA**	0.06	0.10	0.26	0.07	0.31
**Mansfield-Ashland-Bucyrus, OH**	0.06	0.05	0.15	0.05	0.06
**Myrtle Beach-Conway, SC-NC**	0.06	0.04	0.16	0.05	0.11
**Springfield-Jacksonville-Lincoln, IL**	0.06	0.06	0.20	0.06	0.05
**Greenville-Kinston-Washington, NC**	0.06	0.06	0.15	0.05	0.01
**Redding-Red Bluff, CA**	0.06	0.04	0.30	0.03	0.09
**Davenport-Moline, IA-IL**	0.06	0.08	0.25	0.04	0.24
**Gainesville-Lake City, FL**	0.06	0.05	0.22	0.04	0.03
**Morgantown-Fairmont, WV**	0.06	0.04	0.13	0.04	0.09
**Ithaca-Cortland, NY**	0.05	0.04	0.28	0.11	0.02
**Bloomington-Pontiac, IL**	0.05	0.04	0.18	0.04	0.09
**Parkersburg-Marietta-Vien0, WV-OH**	0.05	0.04	0.13	0.07	0.17
**Jonesboro-Paragould, AR**	0.05	0.05	0.14	0.05	0.01
**Bowling Green-Glasgow, KY**	0.05	0.04	0.22	0.08	0.03
**Joplin-Miami, MO-OK**	0.05	0.04	0.16	0.04	0.10
**Tyler-Jacksonville, TX**	0.05	0.07	0.19	0.05	0.00

**Jackson-Brownsville, TN**	0.05	0.05	0.10	0.04	0.05
**Columbia-Moberly-Mexico, MO**	0.05	0.04	0.15	0.01	0.04
**Amarillo-Pampa-Borger, TX**	0.05	0.06	0.27	0.03	0.00
**Lafayette-West Lafayette-Frankfort, IN**	0.05	0.05	0.20	0.05	0.01
**Burlington-South Burlington-Barre, VT**	0.05	0.05	0.05	0.05	0.09
**Paducah-Mayfield, KY-IL**	0.04	0.04	0.10	0.03	0.08
**Altoona-Huntingdon, PA**	0.04	0.06	0.24	0.06	0.00
**Fargo-Wahpeton, ND-MN**	0.04	0.03	0.24	0.03	0.00
**New Bern-Morehead City, NC**	0.04	0.04	0.16	0.06	0.03
**Bloomington-Bedford, IN**	0.04	0.04	0.15	0.04	0.00
**Hattiesburg-Laurel, MS**	0.04	0.04	0.13	0.04	0.01
**Tupelo-Corinth, MS**	0.04	0.03	0.06	0.03	0.02
**Rocky Mount-Wilson-Roanoke Rapids, NC**	0.04	0.05	0.15	0.06	0.01
**Albuquerque-Santa Fe-Las Vegas, NM**	0.04	0.09	0.42	0.00	0.00
**Medford-Grants Pass, OR**	0.04	0.02	0.14	0.02	0.00
**Mount Pleasant-Alma, MI**	0.04	0.03	0.08	0.04	0.03
**Wausau-Stevens Point-Wisconsin Rapids, WI**	0.04	0.03	0.12	0.06	0.00
**Kokomo-Peru, IN**	0.03	0.03	0.12	0.05	0.00
**Eau Claire-Menomonie, WI**	0.03	0.03	0.14	0.02	0.04
**Corpus Christi-Kingsville-Alice, TX**	0.03	0.10	0.35	0.02	0.33
**El Paso-Las Cruces, TX-NM**	0.03	0.07	0.41	0.06	0.00

**Victoria-Port Lavaca, TX**	0.03	0.02	0.18	0.00	0.09
**Cape Girardeau-Sikeston, MO-IL**	0.03	0.03	0.13	0.03	0.06
**Columbus-West Point, MS**	0.03	0.03	0.11	0.04	0.06
**Mankato-New Ulm, MN**	0.03	0.03	0.11	0.03	0.00
**Rochester-Austin, MN**	0.03	0.04	0.12	0.02	0.02
**Edwards-Glenwood Springs, CO**	0.03	0.02	0.20	0.04	0.00
**State College-DuBois, PA**	0.03	0.03	0.13	0.03	0.00
**Williamsport-Lock Haven, PA**	0.03	0.02	0.24	0.07	0.00
**Quincy-Hannibal, IL-MO**	0.03	0.02	0.17	0.01	0.09
**Dixon-Sterling, IL**	0.03	0.03	0.10	0.03	0.08
**Bend-Prineville, OR**	0.03	0.02	0.10	0.01	0.00
**Richmond-Connersville, IN**	0.02	0.02	0.17	0.06	0.00
**Hot Springs-Malvern, AR**	0.02	0.02	0.09	0.04	0.00
**Cleveland-Indianola, MS**	0.02	0.02	0.14	0.02	0.08
**Pullman-Moscow, WA-ID**	0.02	0.01	0.16	0.01	0.17
**Elmira-Corning, NY**	0.02	0.02	0.18	0.07	0.00
**Scottsboro-Fort Payne, AL**	0.02	0.03	0.05	0.04	0.04
**Johnstown-Somerset, PA**	0.02	0.03	0.11	0.04	0.02
**Martin-Union City, TN**	0.02	0.02	0.05	0.03	0.03
**Burlington-Fort Madison-Keokuk, IA-IL-MO**	0.02	0.02	0.09	0.02	0.09
**DeRidder-Fort Polk South, LA**	0.02	0.01	0.06	0.03	0.01

**Moses Lake-Othello, WA**	0.02	0.01	0.12	0.01	0.00
**Rapid City-Spearfish, SD**	0.01	0.02	0.14	0.00	0.00
**Marinette-Iron Mountain, WI-MI**	0.01	0.01	0.07	0.02	0.04
**Spencer-Spirit Lake, IA**	0.01	0.01	0.03	0.01	0.00
**Kerrville-Fredericksburg, TX**	0.01	0.01	0.07	0.00	0.00
**Clovis-Portales, NM**	0.01	0.01	0.21	0.02	0.00
**Steamboat Springs-Craig, CO**	0.00	0.01	0.26	0.01	0.00

Section S1: Discussion of assumptions for race/ethnic asthma prevalence

In estimating the race/ethnic-specific asthma prevalence caused by NO_x emissions, we made two key assumptions: 1) that, for a single Census tract, the baseline asthma rate is uniform across race/ethnicity, and 2) that risk ratio is constant across race/ethnicity. The first assumption is a necessary one; as far as the authors are aware, there does not exist a similarly high spatial resolution dataset with race/ethnicity-stratified baseline asthma rate. Additionally, because Census tracts are relatively small in size and tend to align with neighborhoods in larger urban areas, tracts are more likely to be racially homogenous, such that the asthma rate for a Census tract will be representative across race/ethnicities. The second assumption is also necessary, as there is no evidence in the literature yet for a different risk ratio across race/ethnicities. There is some evidence for a difference in risk ratio for $PM_{2.5}$-related premature mortality by race/ethnicity, suggesting that a similar difference could exist for the risk ratio for NO_2-related asthma prevalence, but this is outside the scope of our work.

Section S2: Discussion of distributions for uncertainty analysis

In this section, we provide additional detail on the distributions we used for Monte Carlo analysis. For population and baseline pediatric asthma rate, we sampled from distributions in the literature. The margin of error on population tends to be very large relative to the value of population itself; across all Census block groups in the U.S., the margin of error is on average 90% of the value of the population, suggesting relatively large uncertainty in population. On the other hand, the distribution of baseline pediatric asthma rate for a given Census tract is relatively small, on average plus or minus two percentage points. Ultimately, neither of these significantly contributes to overall uncertainty in asthma prevalence, as demonstrated by our uncertainty analysis.

For the uncertainty in concentration, we perturbed the predicted concentrations according to a logistic function. First, we sampled two parameters: the "cutoff distance" d, i.e. the point at which the model goes from underpredicting to overpredicting the concentration, which is sampled uniformly from the range [2000, 5000] m; and the perturbation amount p, the amount that the model over/underpredicts concentrations, which is sampled from uniformly from the range [0, 0.5]. These parameters are input into Equation S1, where C is the perturbed concentration, C_{model} is the model-predicted NO_2 concentration, and r is the distance from the source.

$$C = C_{\text{model}} \cdot \left(2p \cdot \left(\frac{1}{1 + e^{-(r-d)/500}} - 0.5\right) + 1\right) \quad \text{(S1)}$$

Section S3: Sensitivity of results to ozone concentration

In this section, we detail the sensitivity analysis we performed with respect to ozone concentration. The ambient ozone concentration is a key input to AERMOD to predict NO/NO_2 interconversion. In the baseline results we present in the main text, we used the seasonal ambient ozone concentration from the nearest ozone monitor to the source to predict marginal asthma prevalence from each source. To examine the sensitivity of marginal asthma prevalence to ozone concentrations, we selected 50,000 source locations across the U.S. and calculated the marginal asthma prevalence under ozone concentrations ranging from 1 to 150 ppb. For ease of comparison across source locations, we normalize the predicted marginal asthma prevalence for a given source location and ozone concentration by the values at that source location at an ozone concentration of 10 ppb.

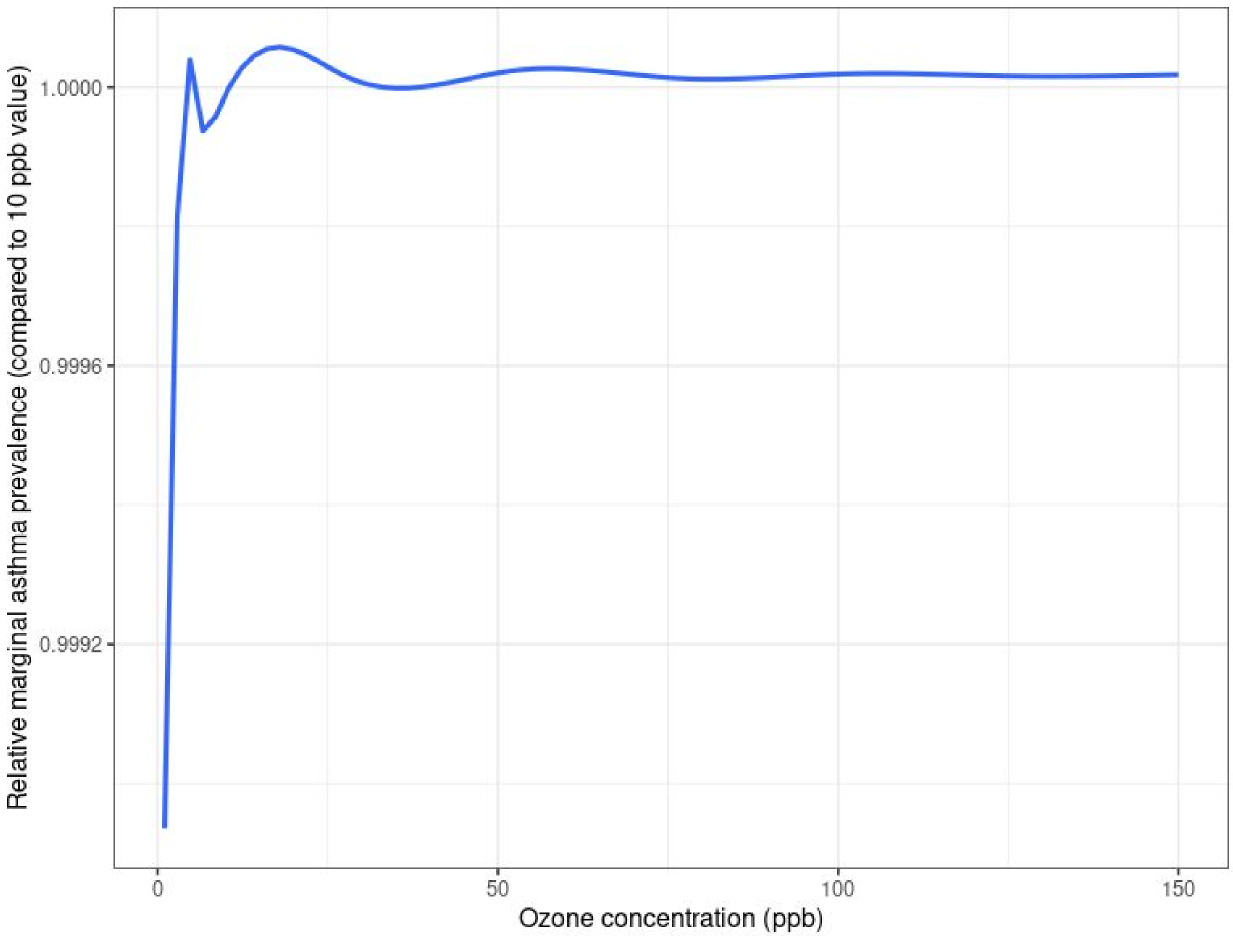

Figure S2: Sensitivity of predicted marginal asthma prevalence to ozone concentration.

Figure S2 presents the normalized marginal asthma prevalence at ozone concentrations from 1 to 150 ppb for 50,000 source locations across the U.S. The marginal asthma prevalence value demonstrates very little sensitivity to the assumed ambient ozone concentration, varying by less than 0.1% over the range of ozone values considered. Our results are robust with respect to our treatment of ambient ozone concentration.
